# Thinking about Enhanced Permeability and Retention Effect (EPR)

**DOI:** 10.3390/jpm12081259

**Published:** 2022-07-30

**Authors:** Stefano Leporatti

**Affiliations:** CNR Nanotec-Istituto di Nanotecnologia, Via Monteroni, c/o Campus Ekotecne, 73100 Lecce, Italy; stefano.leporatti@nanotec.cnr.it; Tel.: +39-083231829

This invited editorial article aims at reporting progress about the enhanced permeability and retention effect (EPR for short), starting from a recent Special Issue in the *Journal of Personalized Medicine* (namely, “EPR Effect-Based Tumor Targeted Nanomedicine”) and focusing specifically on one of these contributing articles, a review from Jun Wu entitled “The Enhanced Permeability and Retention (EPR) Effect: The Significance of the Concept and Methods to Enhance Its Application”, which has recently acquired the rank of a highly cited paper [1]. 

The year 2021 was the 35th anniversary of the EPR effect’s discovery when the first original paper of Matsumura and Maeda in *Cancer Research* was published in 1986 [2]. The EPR effect is a universal patho-physiological phenomenon and mechanism in which macromolecular compounds beyond a specific size can progressively accumulate in the tumor vascularized area and, in this way, carry out targeted delivery and retention of anticancer compounds in the solid tumor tissue [3]. It is believed that the EPR is an effective modality for cancer targeted delivery of a number of macromolecular compounds and drug-loaded nanocarriers [4]. In fact, it has been shown that tumor vessels are highly permeable to macromolecular compounds. After entering tumor tissue, these compounds are entrapped inside the tumor tissue for a prolonged period. Unfortunately, the development of nanomedicine has been hampered for decades in achieving therapeutic advantages in clinical practice. Actually, the existence and intensity of the EPR effect in human solid tumor circumstances have hardly been discussed [5,6]. The EPR effect has also been reported in solid tumors of animals and human patients [1,2,3,5]. The delivery efficiency of nanoparticles into human tumor tissue is very low compared to that in the animal tumor mode. Moreover, the extravasation mechanism for nanoparticles into tumors occurs through tumor vasculature gaps, and organelles [4]. 

In his article, Jun Wu [1] brilliantly overviewed the advantages, pitfalls, and caveats of the EPR effect. In fact, he pointed out a few significative characteristics of tumors where the EPR effect can act more efficiently: (1) strong irregular neovascularization in tumors with abnormalities in tumor blood vessels; (2) elevated expression of inflammatory factors; (3) lack of efficient drainage of lymphatic systems in solid tumor tissue. Among the advantages worthy of consideration is the role of fluid pressure and solid stress in solid tumors. The latter is present due to tumor mass expansion against external normal tissue. In his article, Wu reported that the interstitial fluid pressure or solid stress hampers drug penetration into the center of the tumor tissue, but it does not pose as an obstacle to macromolecular agents in extravasating and accumulating in the peripheral area of the tumor tissue [1]. Thereby, in the peritumoral area, the EPR effect principally occurs. Furthermore, the interstitial fluid pressure and a heterogeneous blood supply are both observed in rodent and human solid tumors [1]. For this reason, a tumor can be suppressed even if an anticancer drug is not penetrating its center, and the EPR effect acting in the peritumoral area is sufficiently successful for reducing or eliminating the neoplasia. The main issue in the use of nanomedicinal tools triggered by the EPR is probably related to the design of the nanocarriers. For example, PEGylation has been shown to have a significative influence in Doxil-loaded liposomes [7]. In fact, PEGylated liposomal doxorubicin exhibits a better therapeutic benefit than that of free doxorubicin. Nevertheless, the overall therapeutic result is not totally satisfactory. The problem of therapeutic efficacy is probably caused by compromised tumor-cell-killing properties by the PEGylation of liposomes. Therefore, it is not the EPR effect that failed in clinical trials, but rather the PEGylation of liposomal chemo drugs that failed to achieve satisfactory cytotoxicity efficacy within a 48 h period in clinical application [7]. Another issue still to solve is the abnormality of tumor blood vessels which impedes the blood to flow into tumor tissue. When attempting to normalize tumor vessels for enhancing the delivery of nanomedicine, the size of nanoparticles, the timing order of drug administration, and vascular normalization are essential factors in reaching the desired delivery results [8]. 

Overall, the design of nanomedicine should be improved for augmenting the EPR effect. Size, surface charge, and physico-chemical properties are key factors for drug-loaded nanocarriers to reach the target by the EPR. Nanoparticles with a size of 100-200 nm are thought to be optimal for achieving a maximized EPR effect [9]. NPs’ surface charge and shape are also very relevant. Negative or neutral surface-charged NPs can reach plasma half-lives of longer than several hours in the circulation by accumulating in neoplastic tissues. In a recent review, it was also reported that discoidal, cylindrical, and ellipsoidal-shaped NPs can accumulate more efficiently in tumors [10]. Furthermore, the delivery of macromolecular drugs can be enhanced by modulating the EPR effect in the targeted tumor tissues by applying adjuvants, inflammatory factors, or antibody photosensitizers [11,12,13,14]. 

Wu also evidenced another critical point for nanomedicine delivery in solid tumors through the EPR: blood flow. Usually, great differences are seen in mice compared to humans: the blood flow rate is roughly 800-fold higher in human normal organs than in mouse normal organs. This difference may be important to explain why nanomedicines can accumulate better in rodent solid tumors than in human solid tumors. Therefore, the application of angiotensin II could be very efficient for drug delivery via increasing the blood flow into stagnated tumor blood vessels [1]. Connected to this point is the fact that intravenous administration of nanomedicines could be problematic in achieving the desired amount of drug in the tumor tissue because of the high shear force to the endothelial wall caused by the fast blood flow. 

Lastly, the selection of animal models for the preclinical development of nanomedicine is also a key issue. Most nanodrugs are developed in small rodent tumor models. The tumors are induced by carcinogens or by knocking in or knocking out certain genes for initiating tumors. Unfortunately, there is a great difference in size between mice and humans, and thus drug absorption, metabolism, distribution, and pharmaco-kinetic/dynamic properties of the drugs in the tumor tissue are very different in mice and humans, leading to a weakening of effective animal models for human applications. 

In summary, the take-home messages one may obtain from this article are the following:

1. The EPR effect is a key principle for designing nanomedicinal tools for targeted and selective drug delivery;
2. To improve the therapeutic efficiency of nanomedicine by the EPR, one may select the optimal shape, charge, and size and design a molecular affinity with tumoral cells through specific functionalization;
3. The blood flow plays a specific role in delivering nanocarriers via the EPR, and, thereby, enhancers could be added to strengthen the therapeutic efficacy;
4. The selection and choice of companion animal models for enhancing nanodrug developments in EPR delivery are recommended.
5. Improving retention efficacy by increasing the concentration and time in tumor tissues is also necessary.
